# Validating a forced-choice method for eliciting quality-of-reasoning judgments

**DOI:** 10.3758/s13428-023-02234-x

**Published:** 2023-10-13

**Authors:** Alexandru Marcoci, Margaret E. Webb, Luke Rowe, Ashley Barnett, Tamar Primoratz, Ariel Kruger, Christopher W. Karvetski, Benjamin Stone, Michael L. Diamond, Morgan Saletta, Tim van Gelder, Philip E. Tetlock, Simon Dennis

**Affiliations:** 1 Centre for the Study of Existential Risk, University of Cambridge, 16 Mill Lane, Cambridge, CB2 1SB UK; 2 Melbourne School of Psychological Sciences, University of Melbourne, Melbourne, Australia; 3 School of Education, Australian Catholic University, Melbourne, Australia; 4 Hunt Laboratory for Intelligence Research, University of Melbourne, Melbourne, Australia; 5 Good Judgment Inc, New York, NY USA; 6 Wharton School, University of Pennsylvania, Philadelphia, PA USA

**Keywords:** Reasoning, Quality of reasoning, Comparative judgment, Forced choice, Automatic reasoning assessment

## Abstract

In this paper we investigate the criterion validity of forced-choice comparisons of the quality of written arguments with normative solutions. Across two studies, novices and experts assessing quality of reasoning through a forced-choice design were both able to choose arguments supporting more accurate solutions—62.2% (SE = 1%) of the time for novices and 74.4% (SE = 1%) for experts—and arguments produced by larger teams—up to 82% of the time for novices and 85% for experts—with high inter-rater reliability, namely 70.58% (95% CI = 1.18) agreement for novices and 80.98% (95% CI = 2.26) for experts. We also explored two methods for increasing efficiency. We found that the number of comparative judgments needed could be substantially reduced with little accuracy loss by leveraging transitivity and producing quality-of-reasoning assessments using an AVL tree method. Moreover, a regression model trained to predict scores based on automatically derived linguistic features of participants’ judgments achieved a high correlation with the objective accuracy scores of the arguments in our dataset. Despite the inherent subjectivity involved in evaluating differing quality of reasoning, the forced-choice paradigm allows even novice raters to perform beyond chance and can provide a valid, reliable, and efficient method for producing quality-of-reasoning assessments at scale.

## Introduction

When eliciting judgments about an unknown quantity, such as the quality of a written argument, one can prompt participants either to directly score an item (cardinal measurement) or to make a comparative judgment (ordinal measurement). Cardinal measurements have been extensively employed in measuring quality of reasoning and argumentation, usually supported by the use of a rubric (Jonsson & Svingby, 2007; Brookhart & Chen, 2015). However, scoring argument quality is time-consuming and subject to various cognitive biases, leading to low inter-rater reliability (e.g., Wachsmuth et al., 2017, Toledo et al., 2019, Gretz et al., 2020). In contrast, ordinal measurements are faster and less cognitively demanding on human raters, reducing the risk of bias and variance (Toledo et al., 2019, Gleize et al., 2019). However, they *force* raters to collapse the multiple relevant dimensions on which two written texts often fare differently (for instance, Wachsmuth et al., 2017, found 15 different categories relevant for measuring quality of reasoning) to a coarse binary *choice*. Moreover, ordinal measurements require significantly more (monotonous) judgments to be made (Bramley et al., 1998) leading Verhavert et al. (2018) to state that “one of the most important methodological questions in CJ [comparative judgments] to date is, how can the efficiency (in number of comparisons) of a CJ assessment be increased without affecting the reliability of the final estimates?” (p. 429).

The aim of the current research was to investigate the criterion validity of *forced-choice* comparisons of the quality of written arguments with normative solutions and explore strategies for producing more efficient comparisons.

The two studies we report on below were conducted as part of IARPA’s Crowdsourcing Evidence, Argumentation, Thinking and Evaluation (CREATE) program.^1^ The CREATE program aimed to develop tools facilitating groups of intelligence analysts to write better-reasoned reports. Within CREATE, the Smartly-assembled wiki-style argument marshalling (SWARM) project^2^ (which included AM, MEW, LR, AB, TP, AK, BS, MLD, MS, TvG, and SD) focused on measuring the gains in quality of reasoning brought about by structured writing techniques modeled after the Delphi method as compared to unstructured methods for collaborating. SWARM constructed a corpus of 279 arguments in support of answers to a wide range of reasoning problems with normatively correct solutions (Study 1, Methods section). We instructed participants to choose the better-reasoned rationale out of pairs of these arguments. Study 1 used an MTurk sample, and Study 2 used an expert sample, composed of people with relevant expertise in judging reasoning. Criterion validity assesses whether a measure is positively related to other measures one would expect it to be related to. We investigated the extent to which forced-choice judgments tracked accuracy, team size, and expertise.

We first expected that normatively correct answers would be accompanied by better arguments. Indeed, this is the underlying assumption of deliberating groups as diverse as juries and scientific collaborations. We argue with one another because we expect that “some arguments must be better than others and ‘argument strength’ must have some meaningful connection with truth” (Hahn, 2020), at least when we have all the relevant evidence. Most reasoning tasks included in this study (see Table 1) provided all information required to solve them in their statement. Additionally, participants solved them in groups, allowing them to share hidden and undistributed information and to scrutinize each other’s reasoning, thus improving their prospects of reaching the correct solution.

**Table 1:** Description of problems included in Study 1

Category / Problem type	Problem (abbreviation)	Description (Source) (quadrant of McGrath’s circumplex)	Scoring	No. of rationales	Avg. rationale length (SD)
1. Verbal comprehension (VBC)	Verbal comprehension 1 (VBC_1)	Tests comprehension of written text (GMAT, 2018) (Type 3, QII)	Correct / Incorrect	18	232 (155)
	Verbal comprehension 2 (VBC_2)		Correct / Incorrect	14	129 (61)
2. Geolocation (Geo)	Geolocation 1 (GEO_1)	Asks for the location and time of a given photo (in-house) (Type 1 and 2, QI)	More to less accurate	16	246 (213)
	Geolocation 2 (GEO_2)		More to less accurate	12	196 (208)
	Geolocation 3 (GEO_3)		More to less accurate	14	239 (135)
3. Critical reasoning (CR)	Critical reasoning 1 (CR_1)	Tests ability to critique an argument (GMAT, 2018) (Type 4, QII)	Correct / Incorrect	17	125 (116)
	Critical reasoning 2 (CR_2)		Correct / Incorrect	13	157 (124)
4. Object identification (OID)	Object identification (OID_1)	Participants are required to identify an object (in-house) (Type 5, QIII)	Correct / Incorrect	16	128 (100)
5. Integrative reasoning (IR)	Integrative reasoning (IR_1)	Tests ability to draw the correct conclusions from data (Manhattan Review, 2012) (Type 3, QII)	Correct / Incorrect	17	130 (107)
6. Document identification (DocID)	Document identification (DocID_1)	Participants must correctly identify the source of the text (in-house) (Type 5, QIII)	Correct / Incorrect	15	121 (91)
7. Syllogisms (Syl)	Syllogisms problem (Syl_1)*	Tests ability to identify consequences of deductive syllogisms (Ennis et al., 1985) (Type 3, QII)	Correct / Incorrect	17	184 (103)
	Syllogisms problem (Syl_2)*		Correct / Incorrect		
	Syllogisms problem (Syl_3)*		Correct / Incorrect		
8. Checkers (Che)	White-team checkers (Che_1)	Based on 5 preceding checkers moves, participants need to correctly predict the 6^th^ move based on a real game (in-house) (Type 7, QIV)	More to less accurate	14	194 (121)
9. Logical reasoning (LR)	Logical reasoning 1 (LR_1)	Tests understanding of logical principles (LSAT, 2015) (Type 4, QII)	Correct / Incorrect	17	115 (92)
	Logical reasoning 2 (LR_2)		Correct / Incorrect	9	126 (83)
10. Matrices (Mx)	Raven’s matrices 1 (Mx_1)	A validated test of fluid intelligence and spatial reasoning (Raven, 1998) (Type 3, QII)	Correct / Incorrect	16	80 (48)
	Raven’s matrices 2 (Mx_2)		Correct / Incorrect	13	93 (77)
11. Bayesian problems (Bayes)	Simple probabilistic (Bayesian) reasoning (Bay_1)	Tests capacity to correctly update probabilities based on evidence (Mandel, 2015) (Type 3, QII)	More to less accurate	15	147 (76)
	Complex probabilistic (Bayesian) reasoning (Bay_2)	Tests ability to extract relevant probabilistic information and use it in a Bayes net to update probabilities (Lagnado et al., 2017) (Type 3, QII)	More to less accurate	13	248 (114)
12. Estimation (Est)	Estimation problem (Est_1)	To answer correctly the team must correctly estimate the number of candies in the jar (in-house) (Type 4, QII)	More to less accurate	13	221 (149)

*The three Syl problems were labeled as parts (a, b, and c) of a single problem presented to participants, and they produced a single rationale for this problem set rather than for each part

Second, we expected larger teams to produce answers that were more accurate and better reasoned. Group performance usually improves with increasing group size, especially for problems of moderate difficulty that require understanding of verbal, quantitative, or logical conceptual systems (Laughlin et al., 2002, 2006; Woolley et al., 2010, Trouche et al., 2014). For example, Kosinski et al. (2012) showed that the probability of finding solutions to cognitively complex problems was logarithmically related to the number of group member responses—findings which were replicated by Vercammen et al. (2019). Moreover, structuring group interaction (using a Delphi protocol for instance) is also shown to further improve the accuracy of group judgments (O’Hagan, 2019) and to counter common cognitive biases. We assembled teams ranging from 5 to 21 members. While we did not mandate a minimum level of participation and we observed many idle participants in most teams, we nevertheless expected that, everything else being equal, larger teams would have more active members and produce more accurate answers and better rationales.

Finally, we expected the correlations between objective accuracy and quality of reasoning to be stronger for experts than for novices. Expertise cannot simply be reduced to credentials (Burgman, 2016). It requires intensive training (Ericsson, 2006) and deliberate practice (Ericsson & Lehmann, 1996), and it needs to be elicited in a structured way (Burgman et al., 2011). Our expert sample included individuals with research and teaching expertise in logic and critical thinking who had extensive experience marking student assignments, and we elicited their judgments in a structured way.

## Study 1: Assessing forced choice using novice raters

In Study 1, we measured criterion validity by assessing whether accuracy and team size affected whether a rationale was selected as better reasoned through a forced-choice design. We pre-registered our hypotheses on the Open Science Framework (see https://osf.io/re5ha) using the pre-registration template provided by AsPredicted.org (https://osf.io/m3spx/). We hypothesized that (1) products resulting in more accurate solutions would be associated with rationales that were chosen more often in forced-choice comparisons; and (2) teams with larger numbers of individuals would produce better-justified rationales than teams with smaller numbers.^3^

### Participants

MTurk raters (*N* = 218) completed the Human Intelligence Tasks (HITS)^4^ at the rate of USD 10/hr. Each pair of rationales was evaluated by exactly three raters.

### Materials

**Fig. 1:**
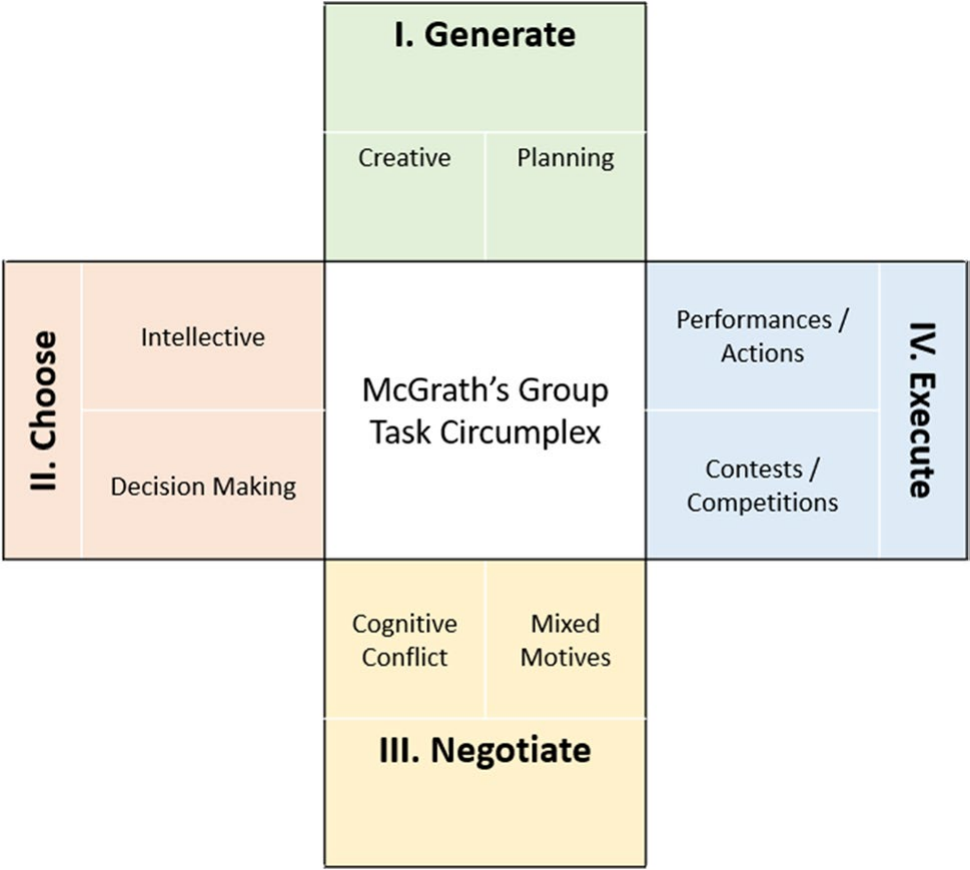
An adaptation of McGrath’s task taxonomy for group tasks (McGrath, 1984, p. 61)

Rationales were produced by teams in the SWARM project. An email invitation was sent to 4179 members of our research pool (van Gelder et al., 2020), of which *N* = 233 consented to participate. They were assigned to teams of varying sizes in two production protocols, and in the end we assembled: four teams of five people, six teams of 10 people, four teams of 15 people, and four teams of 21 people, split evenly across protocols. Participants were given 48 hours (in February–March 2019) to solve 19 problems (Table 1). Two problems, however, were later removed from the dataset as they were mistakenly presented to groups twice (e.g., Logical reasoning 1 and 2 were the same, Raven’s matrices 1 and 2 were the same). The final dataset of problems was based on 17 unique items in 12 different problem categories (Table 1, columns 1 and 2). These were selected to establish a comprehensive sample of different types of collective reasoning tasks that could be completed in a group context. Our item-sampling procedure was guided by prior research that had validated this approach in measuring the general reasoning ability of human groups (see Engel et al., 2014; Riedl et al., 2021; Woolley et al., 2010). These studies drew heavily upon McGrath’s task circumplex, an established group task taxonomy originating from social and organizational psychology, to sample a comprehensive set of tasks based on four qualitatively distinct group processes: generate (create and plan together), choose (analyze and decide together), negotiate (resolve conflicts and competing priorities together), and execute (compete and perform together) (see McGrath, 1984, p. 61). Figure 1 displays an adaptation of McGrath’s group task taxonomy and Table 1 provides specific connections to the item–quadrant combinations we aimed toward; however, we acknowledge that these distinctions are not easily resolved, and overlap from one quadrant to another is inevitable.

Note that some quadrants are more heavily sampled than others as a matter of convenience and context. For example, some of McGrath’s group processes were more easily adapted to our present study context, such as those related to the “judgment” processes, based on the available time and asynchronous communication constraints. The asymmetrical sampling of McGrath’s task circumplex is also evident in the studies that provide precedent for the approach we demonstrate in the present study (e.g., Engel et al., 2014; Riedl et al., 2021; Woolley et al., 2010).

The first production protocol was a simplified version of the Delphi method which uses an iterative cycle of idea generation and consensus building among group members. Delphi methods have been shown to markedly improve “group” performance on forecasting tasks (Hemming et al., 2018; Wintle et al., 2023) by mitigating group biases such as anchoring, group think, and overconfidence. In the first protocol, participants were required to tackle all problems without being able to communicate or share answers with other team members during the first 24 hours. After the initial 24 hours had elapsed, all individual attempts at solving the problems were shared and the team attempted to reach consensus. In the second protocol, participants were given the latitude to solve the problems how they wished and to communicate and share answers with other team members from the outset. Each team submitted a single answer to each problem, though not all teams completed all tasks (and some answers were excluded due to poor quality). For this study we pooled all rationales, irrespective of how they were produced. In total, 279 rationales (avg = 162 words, SD = 132 words) were collected.

### Procedure

Raters were provided with the following instructions:

*A set of complex questions were presented to teams of individuals to solve within 48 hours. Teams were asked to both: 1) Provide the correct answer to each problem, and 2) To provide the background rationale for their answer. In the current HIT, we will 1) Present you with the problems participants were shown, and 2) Ask you to evaluate the reasoning of the answers teams generated. Two pieces of rationale will be presented at the same time: Your task is to decide which team you think justified their answer best by clicking on your preferred rationale.*

**Fig. 2:**
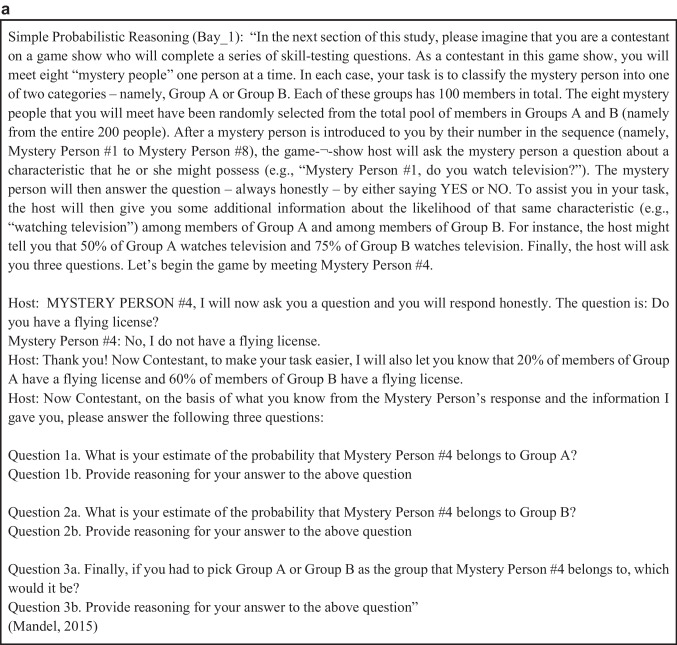

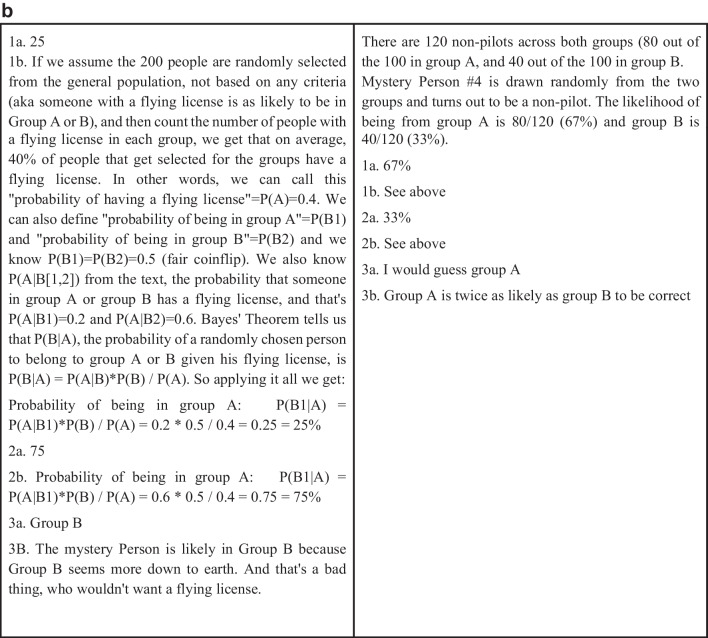
Example of a problem statement (**a**) with two rationales presented side-by-side to raters (**b**)

Raters were then presented with a randomly allocated problem statement (Fig. 2a). Once they had read through the problem statement, raters were presented with two randomly selected rationales corresponding to the problem statement (Fig. 2b) from our corpus of rationales. The rationale that was deemed to be “better justified” was then chosen by the rater. Once the choice was made, they were presented with two more randomly drawn rationales. On average, each rater saw 26.4 pairs of rationales (SD = 31.1). This amounted to a total of 1915 comparisons and choices. Raters were not informed under which production protocol a rationale was generated, what size the team that produced it was, or how accurate the rationale was, and in many cases, both items in a pair were equally accurate. Data collection took place in May 2019.

To assess the relationship between accuracy and the forced-choice measure of quality of reasoning, we first calculated accuracy at the problem level (i.e., Doc_ID_1, GEO_1). Some of the problems in our corpus included multiple questions (see Fig. 2). For each comparison we presented to raters (i.e., team I3’s answer to Bay_1 vs. team I9’s answer to Bay_1), we calculated which team provided more accurate answers to each question (i.e., team I3 or team I9). The team who answered more questions correctly was deemed to have provided a more accurate overall solution to the problem. Once these results were recorded, we were able to combine this information with the results from the forced-choice ratings to assess the probability that a rater would choose a rationale corresponding to a more accurate solution. Answers that were equally accurate were not considered for this analysis.

### Results

1. **Accuracy**. Novices chose the rationale supporting the more accurate solution 62.2% of the time (SE = 1%). See Table 3 for further details.
2. **Comparison between team sizes.** Larger teams produced rationales that were more likely to be chosen compared to teams with fewer members (Table 2). For instance, the probability of MTurk participants choosing a rationale produced by a team of 21 (column) over one produced by a team of 5 (row) was .82 (SD = .02). This corresponds to an effect size of 1.29 (SD = .22, see row 21, column 5 in the MTurk panel of Table 2).
3. **Inter-rater reliability.** The percent agreement between raters was 70.58% (95% CI = 1.18). Chance agreement is 50%, so performance is significantly and substantially better than chance, although far from perfect.
4. **Response time.** While raters must read products upon first presentation, most comparisons were between pairs of products that raters had previously read, and judgments were made quite rapidly. The median reaction time per comparison was just ~9 seconds (mean response time = 29.9 seconds; SD = 100.07). The median response times per problem are outlined in Table 3.

**Table 2:** Bayesian probability estimates of choosing products created by the team with higher numbers of allocated members, by MTurk and expert raters. Below the diagonal line are mean probabilities (and SD); above the diagonal line are effect sizes (and SD). Responses by MTurk raters and expert raters are the left and right halves of the table, respectively

MTurk					Expert				
	21	15	10	5		21	15	10	5
21	-	.40 (.07)	.54 (.15)	1.29 (0.22)	21	-	.43 (.22)	.54 (.15)	1.47 (.24)
15	.61 (.01)	-	.40 (.07)	.87 (.17)	15	.62 (.03)	-	.07 (.14)	.90 (.26)
10	.65 (.02)	.61 (.01)	-	.74 (.16)	10	.65 (.02)	.52 (.02)	-	.78 (.17)
5	.82 (.02)	.73 (.02)	.70 (.02)	-	5	.85 (.02)	.74 (.03)	.71 (.02)	-

**Table 3:** Descriptive statistics by problem for average proportion correct, median response time in seconds, and percentage of forced-choice responses that reflect proximity to the correct answer for MTurk and expert raters

		MTurk		Expert	
Problem	Average proportion correct	Median response time (sec)	Accuracy (SE)	Median response time (sec)	Accuracy (SE)
Overall	0.61	8.97	62.2% (1%)	14.39	74.4% (1%)
Bay_1	0.07	10	52% (3%)	16.25	44% (3%)
Bay_2	0.51	9.5	55% (3%)	-	-
Che_1	0.67	9	52% (3%)	-	-
CR_1	0.78	8	87% (3%)	18.5	96% (2%)
CR_2	0.86	6	36% (6%)	-	-
DocID_1	0.17	8	74% (5%)	-	-
Est_1	0.23	10	51% (3%)	-	-
GEO_1	0.62	9.5	60% (3%)	14	65% (3%)
GEO_2	0.43	10	68% (4%)	-	-
GEO_3	0.57	10	74% (3%)	-	-
IR_1	0.74	7	74% (4%)	14	86% (3%)
LR_1	0.61	10	60% (3%)	9.5	72% (4%)
Mx_1	0.94	6.5	98% (2%)	10.5	93% (5%)
OID_1	0.75	7	65% (5%)	11	90% (3%)
Syl_1	0.9	12	77% (3%)	24.75	92% (2%)
VBC_1	0.75	9	69% (3%)	11	87% (2%)
VBC_2	0.79	11	52% (5%)	-	-

### Discussion

Determining quality of reasoning is inherently subjective and context-dependent (Woods, 2013). Even when provided with detailed guidance, human raters tend to exhibit judgments that have low reliability (e.g., Wachsmuth et al., 2017). Study 1 establishes that a forced-choice design can be used to evaluate quality reasoning. Prompting novice raters to make comparative assessments of reasoning between similar products tends to facilitate valid, reliable, and efficient judgments that align with various dimensions of accuracy. This finding confirms our pre-registered hypothesis that more accurate solutions would tend to be associated with the chosen rationale in a forced-choice comparison.

A written rationale supporting a more accurate solution was significantly more likely to be chosen over a less accurate one, and this trend was relatively strong even among individual raters with no prior training and only minimal guidance. Furthermore, these trends were observed across a wide range of problems with different kinds of reasoning and different levels of difficulty. Indeed, while the proportion of correct answers to the Bay_1 problem was only .07, raters nevertheless selected the Bay_1 rationale supporting a more accurate solution in 52% of cases. For Doc_ID the proportion of correct answers was .17, but raters achieved 74% accuracy (Table 3).

Second, we expected that larger teams would outperform smaller ones. This was reflected in our second hypothesis, which was supported by the results: novices consistently selected the reports generated by larger teams as being better reasoned, amounting to substantial effects (Table 2).

Finally, our secondary analysis found that raters made relatively accurate forced-choice comparisons in a brief amount of time. The median reaction time was ~9 seconds for MTurk participants; however, it should be noted that this trend is not obvious when using the statistical mean because the distribution was highly skewed by the initial reading of the products, which typically takes most participants significantly longer than 9 seconds.

## Study 2: Assessing forced choice using expert raters

In Study 2, we investigated the performance of expert raters with no training and no calibration.

### Participants

“Expert” raters (*N* = 6) were selected on the following criteria: (1) completed or currently completing a postgraduate degree in logic or the psychology of reasoning, and (2) have teaching experience (and had graded coursework) in logic. We recruited five postdoctoral fellows and one advanced PhD student. On average, the experts had 4.83 peer-reviewed articles (SD = 5.27) and taught an average of 13.16 undergraduate courses (SD = 7), 4.66 of which were in logic (SD = 4.36). Raters were compensated at approximately AUD 40/hr. Each pair of rationales was evaluated by two expert raters.

### Methods

The materials, procedure, and measures were as in Study 1, with one exception. For this study, we only investigated a subsample of nine problems that aligned most closely with the area of expertise of the raters (e.g., logic and analytic reasoning) and IARPA-CREATE program goals. Therefore, we sampled one problem from each relevant category (Table 3). Data collection took place in May–June 2019.

### Results

1. **Accuracy.** Experts chose the rationale supporting the more accurate solution 74.4% of the time (SE = 1%). See Table 3 for further details.
2. **Comparison between team sizes.** As in Study 1, we found that larger teams produced rationales that were more likely to be chosen (Table 2); that is, experts were more likely to select using the forced-choice methodology the rationales that were generated by the larger teams. For instance, the probability of experts choosing a rationale produced by a team of 10 (column) over one produced by a team of five (row) was .71 (SD = .02). This corresponds to an effect size of .78 (SD = .17, see row 10, column 5, in the Expert panel of Table 2).
3. **Inter-rater reliability.** The percent agreement between the raters was 80.98% (95% CI = 2.26). As with the novice raters, the reliability is significantly above chance.
4. **Response time.** The response time for comparisons was slightly longer for experts than for MTurk raters (grand median response times were ~14.4 vs. 9 seconds, respectively); however, experts still made their comparisons very quickly (mean response time = 26.77, SD = 40.69). Comparisons of median response time broken down by problem type are presented in Table 3.

### Discussion

Experts chose the product that corresponded to the more accurate answer substantially more often than novices (74.4% as compared to 62.2%). One notable exception was the Bayes network problem (Bay_1), in which accurate solutions were scarce (e.g., .07) compared to other problem types. This not only reduced the number of accurate written products among which QoR could be assessed but may also have undermined the expert raters’ capacity to clearly discriminate better- from worse-reasoned rationales.

The percent agreement is substantially better for experts than for novices (the percent agreement for MTurkers was only 70.58%), as one would expect—adding to the case for the criterion validity of the procedure. However, the difference is perhaps not as large as one might have expected. Agreement depends on the consistency with which raters are addressing the same construct, but also on the discriminability of the choices. It may be that many of our products were not particularly discriminable and that participants were forced to guess. While we adhered to a strict two-alternative choice protocol (as did Toledo et al., 2019, and Gleize et al., 2019), we recognize that other methods, such as those employed by Habernal and Gurevych (2016), gave raters the additional option to say that rationales were equally convincing (e.g., three-alternative methods: better, worse, or equally well reasoned). We suspect such an undertaking would have greatly increased reliability; however, more alternatives may have also led to a trade-off between reliability and efficiency.

In the present study, we observed that experts took longer to make a decision but still made decisions relatively quickly.

We explore the cues participants used to identify better-reasoned rationales in the next section.

## What makes a rationale better reasoned?

In this section we explore the linguistic features of rationales in support of more accurate answers and the differences between rationales selected as better reasoned by experts and MTurkers in the two studies reported above.

We focus on the differences observed between experts and MTurkers in terms of performance, and hence restrict the analysis to the common rationales associated with the nine problems that were rated by both groups. In order to obtain the same number of ratings per rationale, we randomly select two of the three ratings that the MTurkers made per pair (recall each pair was rated by three MTurkers, but only two experts). This sub-corpus includes 148 different rationales, consisting in 1147 different pairwise choices, each rated twice per group, yielding a total of 2294 ratings per group. To calculate the accuracy of each team’s answers and provide comparability across problems for the purposes of the analyses included in this section and the next one, the problem-specific accuracy scores were normalized per problem type by subtracting the mean rationale score and dividing by the standard deviation. The normalized scores were oriented such that lower scores implied greater accuracy. As stated previously, the corpus contains many tied rationales in terms of accuracy of the solutions they support, which were excluded from this analysis. When calculated in this way, in this sub-corpus experts selected the most accurate rationale 76% of the time, whereas the MTurkers did so 66% of the time.

To explore the linguistic features of rationales, we used three different sets of metrics. The first is the set of indicators produced by the Linguistic Inquiry and Word Count (LIWC-22; see Pennebaker et al., 2015)^5^ software, which is the newest version of one of the most popular psycholinguistic tools. The LIWC scores each rationale on over 100 dimensions or categories that include cognition, temporal, emotional, grammatical, and other aspects. Each rationale is scored on each dimension by searching for terms or tokens, and higher scores imply a greater presence of the words or tokens from the category.

The second set of metrics includes the integrative complexity (IC) suite.^6^ IC has been operationalized on a seven-point scale for assessing awareness of alternative perspectives and for connecting perspectives to reach integrative conclusions (see Conway et al., 2020; Conway et al., 2014; Suedfeld & Tetlock, 2014). In addition to general IC, there are two alternative dimensions of IC that are captured by the tool: *dialectical* complexity, which involves grappling with the cognitive tensions between competing perspectives (more usage of “however”), and *elaborative* or cognitive complexity, which involves reducing tensions by generating reinforcing reasons for taking strong stands (more usage of “in addition”; see discussion in Conway et al., 2008).

The last linguistic metric is the comparison class (CC) metric developed by Karvetski et al. (2021). This metric is a crossover metric from the world of geopolitical forecasting, and the model scores highly rationales that feature terms that look for past precedents (e.g., words like “last,” “past”), blending of past data (words like “average”), and comparisons of relativity (“than,” as in “more/less than”).

**Fig. 3:**
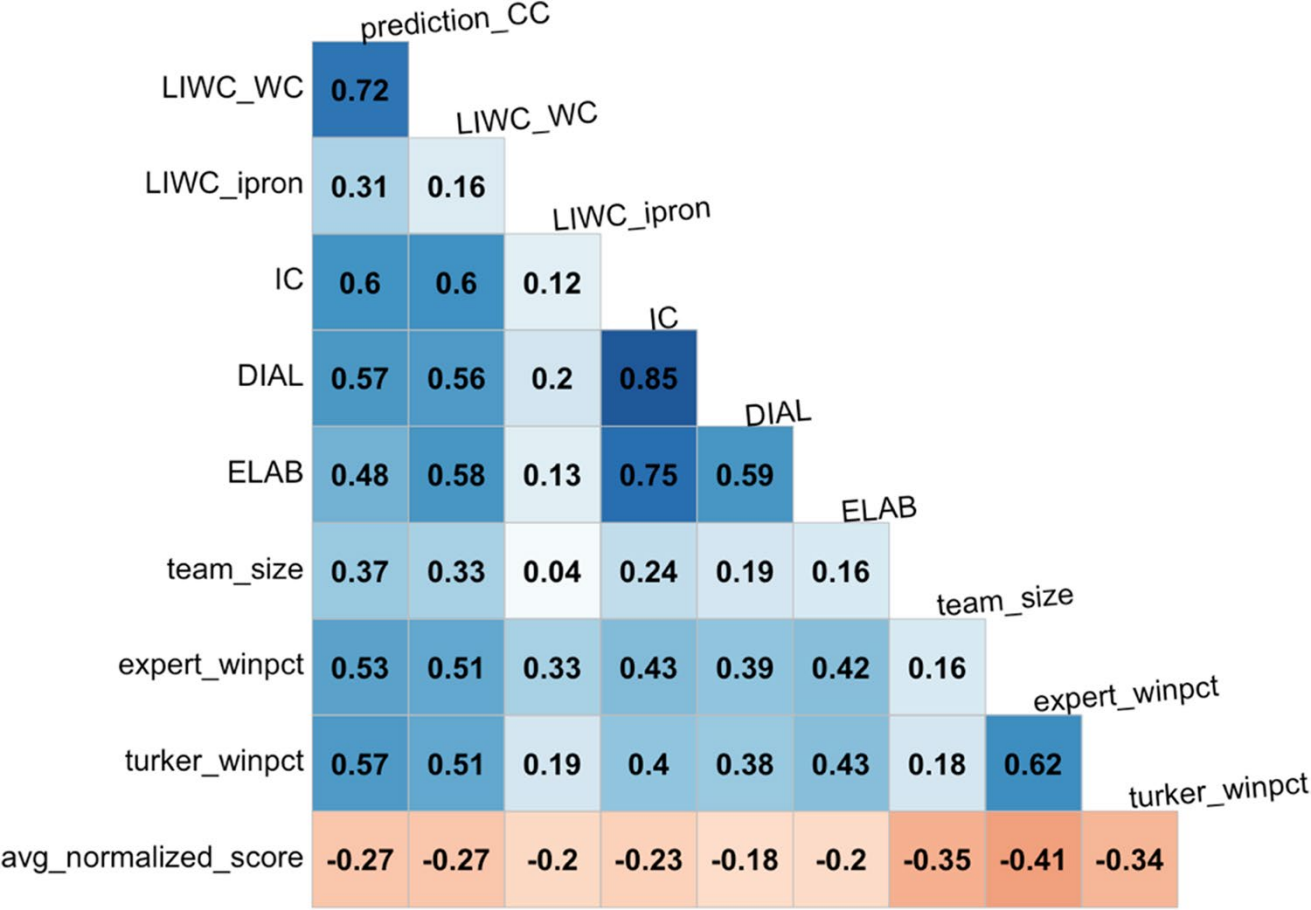
Correlations of linguistic variables and the winning percentage variables with accuracy. All rationales were analyzed using Linguistic Inquiry and Word Count Software, a common psycholinguistic statistical analysis package that allows researchers to examine the relationship between hundreds of variables across a diverse range of written texts (LIWC-22, see https://www.liwc.app/ ); LIWC_WC = Basic Word Count including content-related words (e.g., adjectives, nouns, verbs), function words (e.g., prepositions, conjunctions), symbols such as “p” for probability, numerical expressions such as those used in mathematical formulae, and utterances such as mmm, uh-huh; LIWC_ipron = First Person Pronouns is the frequency by which the written rationale contains first-person pronouns; IC = Integrative Complexity assesses awareness of alternative perspectives and connecting perspectives to reach integrative conclusions; DIAL = Dialectical Complexity involves text demonstrative of opposing views and resolving conflict and contradictions (e.g., “however,” “on the other hand”); ELAB = Elaboration provides text that strengthens argument or extends reasoning using detailed explanations with words such as “moreover” or “additionally”; team_size = the total number of participants in the teams that produced the written rationale; expert_winpct = Winning percentage from all paired ratings of rationales by the expert raters; turker_winpct = Winning percentage from all paired ratings of rationales by the MTurker (non-expert) raters; avg_normalized_score = pertains to the normalized scores for the objective accuracy of problems solved by the groups in relation to their written rationales, noting that all normalization occurred within each of the nine different problem types (within class normalization) by subtracting the mean rationale score and dividing by the standard deviation, with lower scores implying greater accuracy; prediction_CC = Comparison Class is a metric that increases with the proportion of text that looks to past precedents to extend arguments with words like “last” or “previous,” blending with past data with words like “average,” and comparative language such as “more than” or “less than,”

For the below analyses, pairwise choices were transformed to winning percentages. For example, if a rationale won 7 of 10 pairwise matchups across the raters, the winning percentage would be 70%. Figure 3 shows the correlations of winning percentage from all ratings (either “expert_winpct” or “turker_winpct”) with accuracy (“avg_normalized_score”). Also included in Fig. 3 are the variables of team size (“team_size”), and then any variable from the set of 121 linguistic variables that had a correlation of *r* ≥ .3 with these four aforementioned variables. We see that word count, the three IC variables (“IC,” “DIAL” for dialectical complexity, and “ELAB” for elaborative complexity), the comparison class variable (“prediction_CC”), and the use of first-person pronouns (“LIWC_ipron,” i.e., more first-person pronouns) implied better scores, as normalized accuracy is negatively oriented. The strongest accuracy correlate was the expert-derived winning percentage, followed by team size and the MTurker-derived winning percentage. These are medium effect sizes. The MTurkers outperformed word count and the comparison class metric, which were the largest correlates of the linguistic variables.

The two studies described above, and the further exploratory analysis presented in this section, establish that forced-choice assessments of quality of reasoning have high criterion validity and reasonable inter-rater reliability. The results provided by forced choice are consistent between expert and non-expert raters, the protocol itself requires little-to-no training, and the decisions between products can be completed within short time limits (i.e., within a minute). Our results also prove that the method is applicable to longer written products than so far investigated (50–500 in this study compared to, e.g., 8–36 in Toledo et al., 2019). Moreover, the method is context-neutral and could be adapted to evaluate arguments about a variety of topics reliably, especially if supported by additional training for raters (which we have not provided in our studies). However, measuring quality of reasoning through a forced-choice design requires a substantial number of monotonous assessments. In the following two sections we explore strategies for building a more efficient system.

## Efficiency of forced-choice evaluations: Automating assessments

In this section we investigate the accuracy of automated assessments of quality of reasoning performed by a LASSO (least absolute shrinkage and selection operator) regression model trained on a subset of our corpus (described in the previous section).

### Model

Building on the 121 different text-based metrics described in the previous section, we can represent each of the 148 rationales as a unique 121-dimensional vector (with the *i*th rationale represented as *R*_i_). To automate quality of reasoning assessments, we selected LASSO regression (see Hastie et al., 2017, for complete description of the model). The LASSO model is similar to regression but includes a single parameter (*lambda*) that penalizes the sum of the absolute magnitude (i.e., the L1 penalty) of the coefficients. As *lambda* increases, the coefficients “shrink” in magnitude, and some coefficients are zeroed out. Such a procedure has been shown to reduce overfitting and, importantly, leaves the modeler with a subset of coefficients that are nonzero (i.e., the model performs “variable selection”). The optimal *lambda* is found within the internal LASSO cross-validation routine, where *k*-fold cross validation is utilized with training and hold-out data. The value of lambda that minimizes average error on the hold-out sample (known as *lambda.min*) can be utilized to make out-of-sample predictions and to derive the corresponding coefficients on each variable.

### Data and sampling

Similar to the last section, we converted the pairwise choices to a continuous outcome variable by calculating the “winning percentage” (how often a rationale wins the pairwise faceoff) of each rationale within a problem set. In this way, all rationales received a winning proportion score that ranges from 0 (worst) to 1 (best). With these percentages as our dependent training variable, the predictor variables are the 121-dimensional profile vectors.

We performed the modeling exercise twice. First, we randomly selected a subset of the 148 rationales *across* the different problems for training, with the remainder as the testing data, implying that problems featured rationales in both the testing and training datasets, and this training/testing regime highlighted learning of quality of reasoning within the same domains, but on new rationales. Second, we randomly selected all rationales for a given subset of problems, with the complementary problem rationales as the testing data, with an interest in transfer of reasoning. This implied that a problem’s rationales were either all in the training dataset or all in the testing dataset. For both modeling exercises, we tracked the number of nonzero LASSO coefficients selected, the correlation between the model-predicted win proportion and the win proportion derived from the raters (on the testing data), and the correlation of the model-predicted win proportion with normalized accuracy (again, on the testing data). We varied the testing versus training data sample sizes to show convergence as more data were included in the training data sample. Also, we ran the routine 100 times for each sampling size and averaged the performance metrics to reduce simulation noise.

### Results

**Fig. 4:**
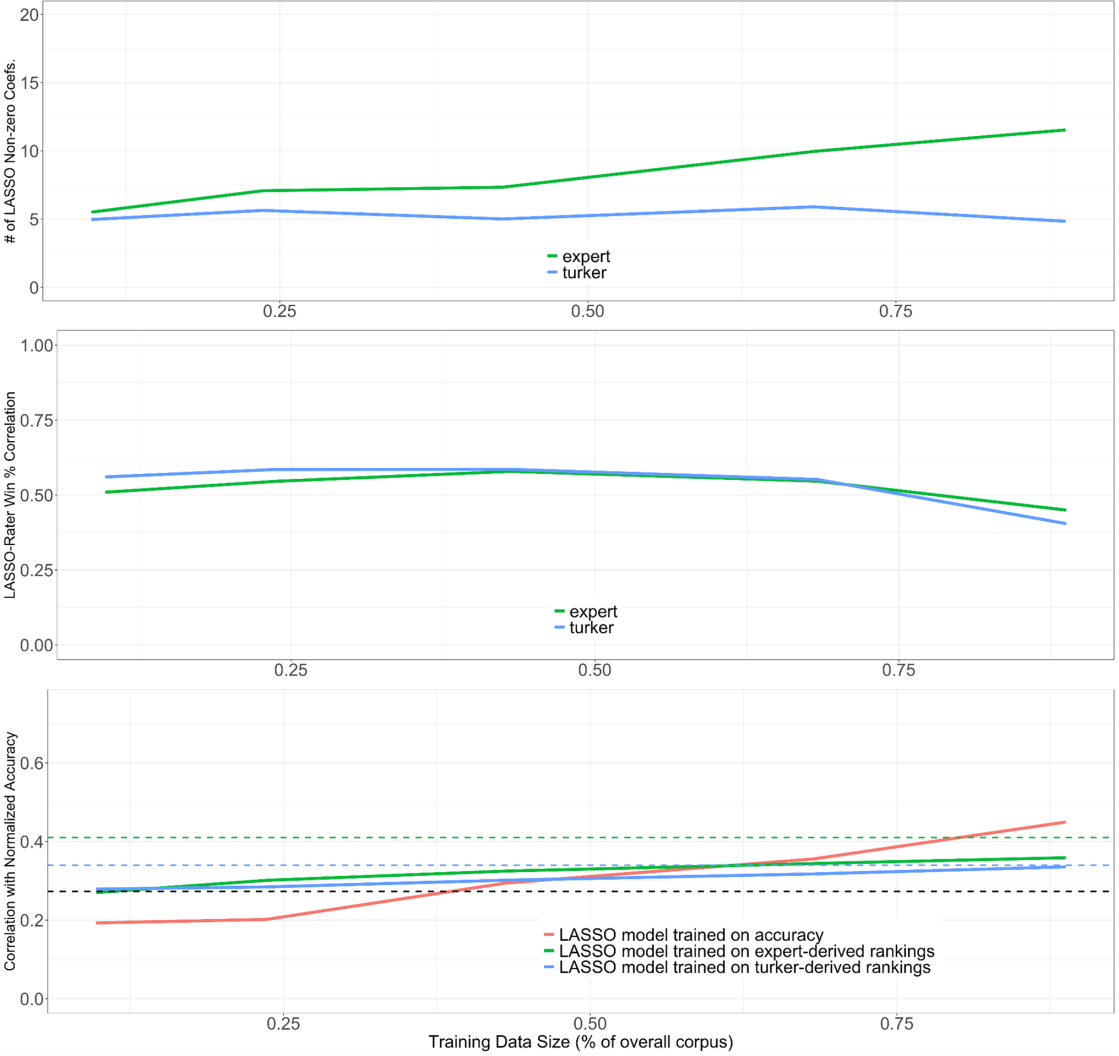
Results of training a LASSO model on mutually exclusive ratings. In panel 3, the black dashed line shows the correlation of word count with normalized accuracy. The green and blue lines show the correlation of normalized accuracy with the rankings derived from expert and MTurker assessments, respectively

We first randomly sampled from the 148 rationales. In Fig. 4, the top panel shows the number of nonzero LASSO coefficients. The second panel shows the correlation of model-predicted winning percentage (when trained on the training data) with realized winning percentage from the testing data, which are large effects, *r* > .5. We see that the correlation drops off at the end, likely because the testing set shrank as the training data increased, and winning percentages were calculated over fewer choices and thus became less stable. The third panel shows correlation with normalized accuracy (of the testing data) when a model was trained on expert-derived winning percentages of the training data (green line), when a model was trained on the MTurker-derived winning percentages of the training data, and when a model was trained on the actual winning percentages of the training data. The green dashed line shows the correlation benchmark of the overall expert-derived winning percentages with normalized accuracy (from *r* = .41, see Fig. 3), the blue dashed line shows the same for the MTurkers (*r* = .34), and the black dashed line shows the correlation of word count with normalized accuracy (*r* = .27).

We see that as data accumulated in the training set, the accuracy-trained model correlated the best with normative accuracy, and that there was little separation between the models trained on expert- and MTurker-derived winning percentages. Furthermore, the fact that the models have a higher correlation with accuracy than word count alone implies that the model is tracking some substantial dimension of reasoning quality. Table 4 shows the nonzero LASSO coefficients associated with the expert-derived winning percentages using all 148 rationales, Table 5 shows the coefficients associated with the MTurker-derived winning percentages, and Table 6 shows nonzero LASSO coefficients associated with normative accuracy as the dependent variable. We note that all models selected the comparison class (prediction_CC) variable and all have zeroed out word count. While these variables are highly correlated (*r* = .72), the comparison class metric is a preferred variable for predicting accuracy when used alongside the other linguistic variables. The LASSO model trained on expert-derived winning percentage featured a mix of cognitive/reasoning styles (e.g., comparison class/elaboration) and generic emotional (e.g., negative emotion) and grammatical (e.g., first-person pronouns) terms, whereas the LASSO model trained on MTurker-derived winning percentages is the simplest and featured only comparison class and elaboration. The accuracy-trained LASSO model featured a mix of all variable types including those that might be case-specific, such as money and ethnicity. Team size correlated moderately and positively with normalized accuracy on the problem sets (*r* = .35). Moreover, there was a moderate correlation between team size and word count (*r* = .33); however, this did not necessarily translate directly into better quality of reasoning according to the ratings of experts and MTurkers. This is evidenced by significantly weaker correlations between team size and the quality of their written reasoning as judged by experts (*r* = .16) and MTurkers (*r* = .18). This suggests that the line of reasoning that larger groups tend to generate more accurate *and* better-reasoned arguments as a direct function of their greater capacity to generate higher word counts is not supported in our dataset.

**Table 4:** LASSO coefficients for expert-derived win percentage

Variable	Coefficient
(Intercept)	0.29
Comparison class (prediction_CC)	0.29
First-person pronouns, e.g., I, me, mine (LIWC_ipron)	0.01
Memory, e.g., remember, reminiscent (LIWC_memory)	0.65
Negative emotional tones, e.g., bad, argument (LIWC_tone_neg)	0.01
Motion, e.g., arrive, car, go (LIWC_motion)	−0.01
Feeling, e.g., feels, touch (LIWC_feeling)	−0.02
Integrative complexity (IC)	0.01
Elaboration (ELAB)	0.01

**Table 5:** LASSO coefficients for MTurker-derived win percentage

Variable	Coefficient
(Intercept)	0.34
Comparison class (prediction_CC)	0.23
Elaboration (ELAB)	0.01

**Table 6:** LASSO coefficients for model trained on normative accuracy

Variable	Coefficient
(Intercept)	−0.32
Comparison class (prediction_CC)	0.39
First-person plural, e.g., we, us, our (LIWC_we)	−0.03
Conjunctions, e.g., and, but, whereas (LIWC_conj)	0.02
Affective process, e.g., happy, cried (LIWC_Affect)	0.01
Ethnicity, e.g., Hispanic, Jewish (LIWC_ethnicity)	−0.13
Money, e.g., audit, cash, owe (LIWC_money)	0.01
Feeling, e.g., feels, touch (LIWC_feeling)	−0.06
Netspeak, e.g., btw, lol, thx (LIWC_netspeak)	0.14

**Fig. 5:**
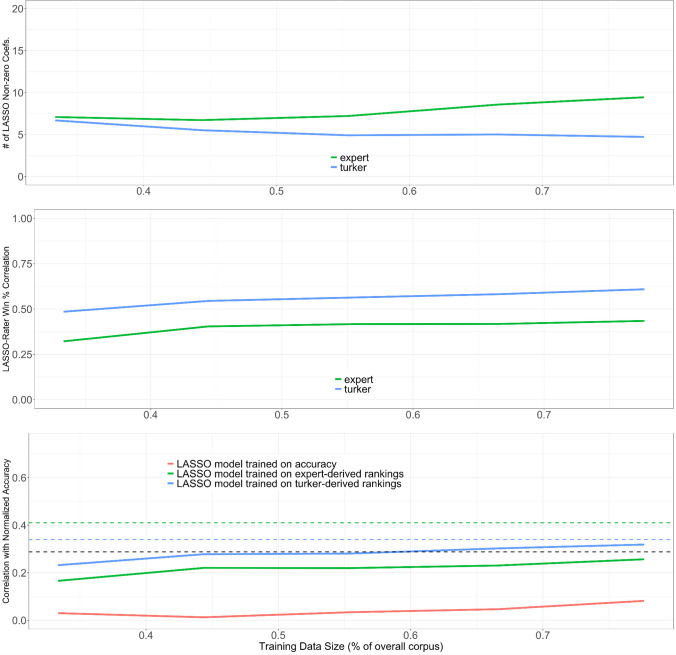
Results of training a LASSO model on mutually exclusive problems. In panel 3, the black dashed line shows the correlation of word count with normalized accuracy. The green and blue lines show the correlation of normalized accuracy with the rankings derived from expert and MTurker assessments, respectively

We then investigated the possibility of transfer of reasoning. The key difference with the previous setup is that the training data were formed by first selecting a subset of the nine problems and then training on all rationales from those problems. This implies there was no crossover of rationales from the same problems for the training and testing data. In Fig. 5, panel 1 shows a similar number of nonzero coefficients as above, but in panel 2, we see that the correlation between LASSO-predicted winning proportion and that derived from the experts’ choices was lower than the comparable correlation of the MTurkers. In other words, the model-assessed reasoning did not transfer as well to other problems for the experts as with the MTurkers. Lastly, panel 3 shows that the models struggle to achieve a correlation with accuracy that was comparable to word count, and accuracy-based training was the worst of the three models, likely because it included more case-specific variables (see Table 6), whereas the Turker-derived model was most likely to transfer to other problems, due to it being the simplest model, featuring only two generic analytic/reasoning variables. Overall, this modeling experiment underscores the challenge of transfer of reasoning.

## Efficiency of forced-choice evaluations: Exploiting transitivity through AVL trees

In this section we explore an alternative to reducing the number of comparisons for generating forced-choice assessments, leveraging the transitivity of our participants’ judgments.

In the studies presented above, and in the previous studies that have used the forced-choice procedure to assess quality of reasoning (e.g., Toledo et al., 2019), all possible combinations of products were compared. For a corpus of size *n*, this means that one must elicit *n*(*n* − 1)/2 judgments. If one wishes to increase the reliability of each ranking, one may require multiple judgments per comparison, which further increases the number of judgments that must be collected. To avoid significant cost, the number of products must be restricted, which is not ideal when one wishes to train reliable classifiers.

However, increased efficiency could be achieved by taking advantage of transitivity. It seems plausible that if product A is rated as better than product B, and product B is rated as better than product C, then one could infer that product A is better than product C without explicitly making that comparison. In our corpus of written rationales, we observed that 73.9% of triplets in the MTurk condition and 90.35% of triplets in the expert condition satisfied transitivity. Toledo et al. (2019) found that transitivity held for 96.2% of all argument triplets in their corpus for which all pairwise combinations were annotated, and similar results were reported by Gleize et al. (2019), who found that 99% of their triplets satisfied transitivity. While the percentage of transitive triplets is lower in our dataset, we believe the discrepancy is explained by our use of longer arguments and by the lack of expertise in the novice (MTurk) condition. Nonetheless, in general, transitivity generally holds and may be useful when considering how to improve the efficiency of data collection by the judicious selection of the pairs to present to raters.

The approach that we chose to test is to add products to an AVL tree (Adelson-Velsky & Landis, 1962). An AVL tree is a self-balancing binary search tree that can be used to create a complete ordering of a set of items. The AVL tree requires only pairwise order comparisons and takes advantage of transitivity to allow insertions into the tree to occur in O(log *n*) comparisons (where *n* is the total number of items to be inserted). Typically, the comparisons would be completed within a program, but in our case, we will have human raters make the comparisons. Comparing every product against every other product takes O(*n*^2^) comparisons, but using the AVL tree this can be reduced to O(*n* log *n*) comparisons, which for large datasets can be a significant saving.

The AVL tree assumes transitivity. Empirically, however, this is only partially true, and so the use of an AVL tree in this way will create some distortion of the ordering of the products. To assess the loss of reliability, we calculated Spearman’s rank correlations between a reference ranking and the ranking produced in each condition. For the MTurk conditions, the reference ranking was created using all three MTurk raters and all comparisons. For the expert conditions, the reference ranking was created using the two expert raters and all comparisons.

Table 7 shows the performance using either MTurk ratings or expert ratings—for each of the problems and averaged across problems. On the left-hand side, we show the correlations for the MTurk raters using all comparisons but only one rater, and then for AVL trees using either three raters or one rater. The correlation using a single rater and all comparisons remains high. With the AVL trees, using three raters yields good performance, but using only one rater introduces significant distortion compared to the reference ordering. A similar pattern emerges for the expert raters. Using one rater with all comparisons or an AVL tree with two raters maintains good performance, but using only a single rater with the AVL trees decreases the reliability of the ranking substantially.

**Table 7:** Correlations between the majority choice for (three) MTurk and (two) expert raters based on assessments of all pairwise comparisons and: the choice of a randomly chosen single rater (all comparisons/one rater) for all comparisons; the majority choice of three MTurk and two expert raters, respectively, when using the AVL tree approach (AVL/three raters); and the choice of a randomly chosen single MTurker and expert, respectively, when using the AVL tree approach (AVL/one rater)

	MTurk			Expert		
	All comparisons	AVL		All comparisons	AVL	
Problem	One rater	Three raters	One rater	One rater	Two raters	One rater
Average	0.92	0.91	0.58	0.88	0.94	0.47
OID_1	0.95	0.98	0.68	0.81	0.92	0.52
LR_1	0.94	0.91	0.74	0.96	0.97	0.41
Bay_1	0.95	0.86	0.48	0.88	0.94	0.19
Geo_1	0.95	0.91	0.84	0.85	0.96	0.91
Mx_1	0.81	0.89	0.44	0.9	0.96	0.24
VBC_1	0.89	0.96	0.56	0.9	0.99	0.69
IR_1	0.97	0.95	0.72	0.87	0.92	0.68
Syl_1	0.94	0.86	0.38	0.88	0.94	0.17
CR_1	0.89	0.84	0.36	0.87	0.85	0.44

Using one rater and all comparisons or AVL trees and three novice comparisons produces approximately equivalent results. However, the number of comparisons required is quite different, particularly as the number of products to be rated increases. For instance, with 100 products, performing all comparisons once requires 4950 ratings, while using the AVL tree requires about 1993 ratings. If one has 1000 products, however, collecting all comparisons requires 499,500 ratings, while the AVL tree approach requires only about 29,897 ratings. The savings in terms of ratings required can be substantial.

## Conclusion

Assessing quality of reasoning is challenging. Most prior work has relied on rating scales which are compromised by both inter- and intra-rater variability. In this paper, we test a forced-choice procedure that eliminates these problems. To establish criterion validity, we show that endorsement in a forced-choice judgment is associated with rationales supporting more accurate answers, made by larger teams and made by people with higher levels of expertise. We also explored two methods for reducing the burden of generating large numbers of pairwise comparisons. The first involved training a regression model to predict scores based on automatically derived linguistic features. While the method works well within domain, more work is required to understand under what conditions it can be accurately applied across problem domains. Second, we found that the intelligent selection of comparisons to present to raters using AVL trees can substantially decrease the number judgments required while maintaining high accuracy. When coupled with the remarkable speed with which raters can make judgments, it suggests that forced choice is a valid, reliable, and efficient method for measuring quality of reasoning in written arguments.

## Data Availability

The datasets generated and/or analyzed are available on OSF, https://osf.io/9qhxn/files/. Except for the in-house problems, all other problems analyzed in this study are available from the third parties identified in the bibliography, but restrictions may apply to their availability. Therefore, items not included in these supplementary materials can only be accessed with permission from the licensor.

## Footnotes

1 https://www.iarpa.gov/research-programs/create.

2 https://www.imperial.ac.uk/security-institute/research/data-processing-and-algorithms/swarm/.

3 We pre-registered a third hypothesis regarding the difference in accuracy between production protocols. This does not have any bearing on assessing the criterion validity of a quality of reasoning measure, and we do not report on it in this paper. We nevertheless describe the two production protocols below.

4 A Human Intelligence Task, or HIT, is a question that needs an answer on Amazon’s Mechanical Turk platform.

5 https://www.liwc.app/.

6 https://www.autoic.org/.

## Code availability

The code used is available on OSF, https://osf.io/9qhxn/files/.
